# Distinct patterns of apolipoprotein C-I, C-II, and C-III isoforms are associated with markers of Alzheimer’s disease

**DOI:** 10.1194/jlr.RA120000919

**Published:** 2020-12-18

**Authors:** Yueming Hu, Cristiana Meuret, Ashley Martinez, Hussein N. Yassine, Dobrin Nedelkov

**Affiliations:** 1 Isoformix Inc, Phoenix, Arizona, USA; 2 University of Southern California, Los Angeles, California, USA

**Keywords:** apolipoproteins, apoC-I, apoC-II, apoC-III, apoE, amyloid-β42, CSF, mass spectrometry, proteomics, plasma, AD, Alzheimer's disease, BBB, blood-brain barrier, BCB, blood-cerebrospinal fluid barrier, CVD, cardiovascular disease, DPP-IV, dipeptidyl peptidase-IV, H/D, hydrogen/deuterium, PBS, phosphate buffered saline buffer, TG, triglyceride

## Abstract

Apolipoproteins C-I, C-II, and C-III interact with ApoE to regulate lipoprotein metabolism and contribute to Alzheimer's disease pathophysiology. In plasma, apoC-I and C-II exist as truncated isoforms, while apoC-III exhibits multiple glycoforms. This study aimed to *1*) delineate apoC-I, C-II, and C-III isoform profiles in cerebrospinal fluid (CSF) and plasma in a cohort of nondemented older individuals (n = 61), and *2*) examine the effect of APOE4 on these isoforms and their correlation with CSF Aβ42, a surrogate of brain amyloid accumulation. The isoforms of the apoCs were immunoaffinity enriched and measured with MALDI-TOF mass spectrometry, revealing a significantly higher percentage of truncated apoC-I and apoC-II in CSF compared with matched plasma, with positive correlation between CSF and plasma. A greater percentage of monosialylated and disialylated apoC-III isoforms was detected in CSF, accompanied by a lower percentage of the two nonsialylated apoC-III isoforms, with significant linear correlations between CSF and plasma. Furthermore, a greater percentage of truncated apoC-I in CSF and apoC-II in plasma and CSF was observed in individuals carrying at least one *APOE* Ɛ4 allele. Increased apoC-I and apoC-II truncations were associated with lower CSF Aβ42. Finally, monosialylated apoC-III was lower, and disialylated apoC-III greater in the CSF of Ɛ4 carriers. Together, these results reveal distinct patterns of the apoCs isoforms in CSF, implying CSF-specific apoCs processing. These patterns were accentuated in *APOE* Ɛ4 allele carriers, suggesting an association between *APOE4* genotype and Alzheimer's disease pathology with apoCs processing and function in the brain.

Apolipoproteins C-I, C-II, and C-III (collectively termed apoCs) reside on lipoprotein particles where they take part in regulating lipid metabolism and transport. The three apoCs are small proteins: apoC-I is comprised of 57 amino acids, and both apoC-II and C-III contain 79 amino acids. All three apoCs exhibit isoforms in vivo. In addition to the full-length translated and processed protein, apoC-I also exists as a truncated form (apoC-Iʹ), lacking the two N-terminal amino acids (1), which is created by the action of dipeptidyl peptidase-IV (DPP-IV) (2). ApoC-II also undergoes proteolytic cleavage, resulting in the removal of its N-terminal six amino acids and yielding an isoform termed mature apoC-II (apoC-IIʹ) (3). On the other hand, apoC-III is glycosylated at Thr^74^, with the most common glycoforms having an O-linked N-acetylgalactosamine-Galactose disaccharide (-GalNAc-Gal), which can be further modified with up to two sialic acid (Sia) residues (1). All three apoCs are expressed predominantly in the liver, and upon entry into plasma, they are rapidly exchanged among the major classes of lipoproteins where they play an important role in the regulation of triglyceride (TG) metabolism (4, 5, 6).

While much is known about apoCs in plasma, their roles in the central nervous system (CNS) remain obscured. The CNS is separated from the peripheral tissues by the blood-brain barrier (BBB) and blood-cerebrospinal fluid barrier (BCB). CNS contains only HDL-like lipoprotein particles that are typically enriched with apolipoproteins A-I, E, and J (7, 8). The early evidence for the presence of apoC-I in the brain came in the form of apoC-I mRNA from marmoset brain tissues, which was present in the brain at only a fraction (<5%) of the mRNA expressed by the liver (9). Gene microarray data from postmortem prefrontal cortex tissues revealed that apoC-I mRNA is highest within the first 5 years of life, but still at much lower levels than the other highly expressed apolipoproteins in the CNS (10). The gene microarray data also revealed minimum amounts of apoC-II mRNA in the brain that increased in school-age children and then fell back below detection levels toward adulthood; the gene expression values for apoC-III were below the limit of reliable detection at all ages (10). Nevertheless, apoC-II and apoC-III were detected at the protein level in cerebrospinal fluid (CSF) using electroimmunoassays at concentrations that were <5% of their levels in plasma (11). These low CSF levels were confirmed in a recent study of 22 matched plasma and CSF samples that showed apoC-III in CSF to be 0.01% of its concentration in plasma (12). Interestingly, plasma apoA-I-containing apoC-III, but not total plasma apoC-III, correlated most strongly with CSF apoC-III. This suggests possible crossing of apoC-III from plasma into CSF along with apoA-I, which has been postulated to cross the BCB via cellular mediated transport (13) and the BBB through clathrin-independent and cholesterol-mediated endocytosis (14). Crossing of the apoCs from plasma into CNS has also been postulated, but has not been conclusively demonstrated.

*APOE4* is the strongest genetic risk factor for late-onset Alzheimer's disease (AD). *APOE4* influences the expression of the apoCs and how they are biologically processed. The genes for apoC-I and apoC-II are located on the long arm of chromosome 19 (19q13.32), along with the gene for apoE (15). The *APOE* ε4 allele is typically expressed with the *APOC1* allele, H2. Indeed, *APOC1* polymorphisms are associated with *APOE*, and the H2 allele is associated with an increased risk of developing AD (16). Apolipoprotein C-III is separately encoded by a region of the long arm of chromosome 11q23, known as the apoA-I/C-III/A-IV gene cluster (17). As an abundant apolipoprotein in TRL, apoC-III induces hypertriglyceridemia and promotes atherogenesis, and it is an important risk factor for cardiovascular disease (CVD) (18, 19, 20). In one study, *APOE* ε4 carriers had lower TG, ApoE, and ApoC-III levels, and the ApoC-III/ApoE ratio on HDL was reported to be the highest (21). Therefore, understanding how the 4 allele affects the apoCs' expression and their posttranslational modifications may further elucidate the biological mechanisms behind protein-related pathologies, such as AD and CVD. We have recently developed a mass spectrometry (MS)-based assay for detection of apoCs and their isoforms (22), and applied it to study apoC-III plasma glycoforms and how they are associated with clinical lipid measures and outcomes (23, 24, 25, 26). In this work, we have examined for the first time the distribution of the apoCs isoforms in CSF and compared it with paired plasma samples from a cohort of older, nondemented individuals grouped by *APOE* genotypes.

## Materials and methods

### Reagents

Polyclonal goat anti-human antibodies to apoC-I (Cat. No. 31A-G1b), apoC-II (Cat. No. 32A-G2b), and apoC-III (Cat. No. 33A-G2b) were obtained from Academy Biomedical (Houston, TX). Acetone (UN1090) was from JT Baker (Radnor, PA). Hydrochloric acid (HCl; AB06037), trifluoroacetic acid (TFA, AB02010), and acetonitrile (ACN; AB00120) were from AmericanBio (Natick, MA). *N*-methylpyrrolidinone (NMP; BP1172-4), 1,1ʹ carbonyldiimidazole (97%) (CDI, 115533), phosphate buffered saline buffer (PBS, 28372), 2-(*N*-morpholino) ethanesulfonic acid (MES) saline buffer (28390), and Mass Spectrometric Immunoassay (MSIA) Tips (991CUS01) were acquired from Thermo Fisher Scientific (Waltham, MA). Tween20 (Cat. No. P7949), sinapic acid (85429), and ethanolamine (ETA; 398136) were obtained from Sigma Aldrich (St. Louis, MO). ApoC-I (Cat. No. EA8011-1), C-II (EA8012-1), and C-III (EA8133-1) ELISA kits were acquired from Assay Pro (St. Charles, MO).

### Human samples

A set of 61 paired human EDTA plasma and CSF samples were analyzed for the apoCs. Recruitment methods were directed at persons enrolled in the University of Southern California (27) Alzheimer Disease Research Center (ADRC) aged 60 years and older. Inclusion criteria included a Clinical Dementia Rating Scale (CDR) score of 0 (n = 48), 0.5 (n = 9), and 1 (n = 1). The study and procedures were approved by the Institutional Review Board of USC. All participants provided informed consent prior to enrollment in the study (USC IRB: HS-16-00888).

### ApoC-III and apoC-I/C-II MSIA tips preparation

Activation and derivatization of the microcolumns inside the MSIA Tips were performed on a Multimek 96 automated 96-channel pipettor (Beckman Coulter, Brea, CA). The MSIA Tips were first rinsed with 200 mM HCl (10 aspiration/dispense cycles, 100 μL each), followed by water (10 cycles) and acetone (10 cycles). Then, the microcolumns inside the tips were activated with CDI (100 mg/mL in NMP (1,000 cycles, 50 μL each) followed by two rinses with NMP (10 cycles each, 100 μL). For the apoC-III tips, the activated tips were immediately immersed into the wells of a 96-microwell microplate containing 2.5 μg apoC-III antibody/well (in 100 μL of 10 mM MES buffer), and 1,000 cycles (50 μL each) were performed, allowing for antibody attachment to the activated microcolumns. For the apoC-I/C-II multiplex tips, the activated tips were immersed into wells containing 0.32 μg apoC-I antibody and 2.25 μg apoC-II antibody/well (in 100 μL of 10 mM MES buffer). Following the antibodies' attachment, the tips were rinsed with ETA and two rinses with PBS (50 cycles each, 100 μL). The total time taken for activation and derivatization of 96 MSIA Tips was 1.5 h. The apoC-III and apoC-I/C-II antibody-derivatized tips were stored at 4°C until use.

### Analytical samples preparation

Plasma and CSF samples were thawed and diluted immediately prior to running the assays. For plasma analyses, the first dilution (S1) was prepared by mixing 3 μL plasma with 117 μL of PBS, 0.1% Tween (PBST). Then, 40 μL of the S1 dilution was mixed with 120 μL of PBST, yielding 160 μL analytical plasma samples (S2 dilution). Two S2 dilutions were prepared from one S1 dilution: the first S2 was for analysis of apoC-III, and the second S2 was for analysis of apoC-I/C-II. For the CSF analyses, 100 μL of CSF was mixed with 100 μL of PBST. The 200 μL analytical CSF samples were used first for apoC-III assay and then (sequentially) for the apoC-I/C-II assay. Higher volumes of CSF were utilized because the apoCs' concentrations in CSF are much lower compared with plasma.

### Assays execution

The antibody-derivatized tips were mounted onto the head of the Multimek 96 pipettor and first rinsed with PBST (10 cycles, 100 μL). The tips were then immersed into the wells of a microplate containing the analytical samples, and 250 cycles (100 μL each) for plasma, and 500 cycles for CSF, were performed, allowing for affinity capture of the targeted proteins. Then, one rinse with PBST (100 cycles, 100 μL) and two rinses with water (10 cycles each, 100 μL) followed to wash off the nonspecifically bound proteins from the microcolumns. To elute the captured proteins, 5 μL of MALDI matrix (20 g/L sinapic acid in 33% (v/v) ACN and 0.4% (v/v) TFA) was aspirated into each tip, pushed up and down three times, and then dispensed directly onto a 96-well formatted MALDI target. Sample spots were dried on a hot plate at 50°C.

### MALDI-TOF MS detection

Bruker Autoflex III MALDI-TOF instrument (Bruker, Billerica, MA) was utilized to acquire linear mass spectra. The instrument was operated in positive ion mode with 20.00 kV and 18.45 kV ion source voltages. The mass spectra were acquired in the mass range from 5 to 20 kDa, with 50 ns delay, and signal suppression of up to 4,500 Da. Total of 1,000 laser-shots were acquired and summed for each mass spectrum.

### Quantification of human plasma and CSF apoCs

The concentrations of plasma and CSF apoCs were determined by Sandwich ELISAs, performed per manufacturer's instructions and as described (28). CSF apoCs concentrations were low but still above the detection limits of the ELISAs; CSF samples were assayed without any dilutions. Plasma samples were diluted 1:1,000 for the apoC-III ELISA and 1:200 for apoC-I and apoC-II ELISAs.

### Data analysis

The mass spectra were first externally calibrated with protein calibration standards and then internally calibrated using the highest intensity apoCs signals. The spectra were baseline subtracted (Convex Hull algorithm, 0.8 flatness) and smoothed (Savizky Golay algorithm, 5 m/z width and 1 cycle) using Flex Analysis software (Bruker Daltonics). Areas under the peaks for all isoforms of apoC-I, apoC-II, and apoC-III signals were integrated using Zebra 1.0 software (Intrinsic Bioprobes Inc) and tabulated in a spreadsheet. To obtain the percent abundance of truncated apoC-I, the peak area of truncated apoC-I was divided with the summed peak areas of both truncated and full-length apoC-I. The percent abundance of truncated apoC-II was similarly calculated. The percent abundance of the individual apoC-III isoforms was calculated by dividing the peak area of each isoform with the summed peak areas of all apoC-III isoforms.

GraphPad Prism 7 was utilized for statistical analysis. Normality of the data sets was assessed with the Shapiro-Wilk test. To identify apoCs isoforms differences between the paired plasma and CSF samples, parametric *t*-test was performed for normally distributed data sets, and Wilcoxon matched-pairs signed rank test was applied to data sets that were not normally distributed. The correlations between the apoCs isoforms in the paired CSF and plasma samples, between CSF Aβ42 and the apoCs isoforms, and between the apoCs isoforms and the total concentrations of apoCs in both CSF and plasma, were assessed via Pearson's correlation (for parametric data sets) or Spearman's rank correlation (for nonparametric data sets). Individuals were further separated into two groups based on the presence of the *APOE* Ɛ4 allele: non-Ɛ4 carriers and Ɛ4 carriers (heterozygous or homozygous for Ɛ4). The percent abundance of the apoC-I, apoC-II, and apoC-III isoforms in plasma and CSF was compared between these two unpaired groups using an unpaired *t*-test with Welch's correction for the normally distributed data sets and the Mann-Whitney test at 5% false discovery rate for data sets that were not normally distributed. Differences among non-Ɛ4, homozygous Ɛ4, and heterozygous Ɛ4 allele carriers were examined with Kruskal-Wallis test with Dunn's multiple comparisons test for nonnormally distributed data and one-way ANOVA with *post-hoc* Tukey HSD test at a 5% false discovery rate for normally distributed data.

## Results and discussion

The paired human CSF and plasma samples from 61 individuals were analyzed for total apoCs concentrations using sandwich ELISAs and for the isoform ratios using MS-based immunoassays. The MS-based immunoassays were comprised of two steps: *1*) immuno capture of apoCs from the samples using antibodies immobilized within a porous microcolumn inside a pipettor tip, and *2*) elution and detection of the captured intact apoCs with MALDI-TOF MS. The assays are fast (∼1 h) and high-throughput (96 samples at a time). The assays determine the relative abundance of the isoforms as a percentage of the total protein. These types of assays have been developed and employed for analysis of several plasma proteins (29), including other apolipoproteins (30). The apoCs assays were applied to CSF samples for the first time in this study. A summary of the 61 individuals cohort by APOE genotype subgroups and concentrations of the apoCs is shown in Table 1.

**Table 1:** Differences in demographic characteristics, plasma apoCs levels, and CSF apoCs levels among *APOE* subgroups

	E2/E3 (N=5)	E2/E4 (N=2)	E3/E3 (N=27)	E3/E4 (N=16)	E4/E4 (N=11)	Total (N=61)	*P*-value
Age							0.042^a^
Mean (SD)	66.8 (6.4)	73.5 (2.1)	67.7 (6.9)	63.6 (5.6)	61.9 (7.7)	65.7 (7.0)	
Sex							0.241
female	3 (60%)	2 (100%)	17 (63%)	6 (38%)	8 (73%)	36 (59%)	
male	2 (40%)	0 (0%)	10 (37%)	10 (63%)	3 (27%)	25 (41%)	
Ethnicity							0.510
Hispanic/Latinos	0 (0%)	0 (0%)	3 (11%)	4 (25%)	1 (9%)	8 (13%)	
Not Hispanic/Latinos	5 (100%)	2 (100%)	24 (89%)	12 (75%)	10 (91%)	53 (87%)	
BMI							0.700
Median (IQR)	27.4 (4.8)	24.0 (NA)	26.8 (5.7)	25.5 (4.7)	24.4 (3.7)	25.4 (5.5)	
Years of Education							0.658
Median (IQR)	16.0 (1.0)	15.0 (3.0)	17.5 (2.8)	16.5 (4.5)	17.0 (2.0)	17.0 (3.0)	
CDR							0.690
0	4 (100%)	2 (100%)	22 (88%)	12 (75%)	8 (73%)	48 (83%)	
0.5	0 (0%)	0 (0%)	2 (8%)	4 (25%)	3 (27%)	9 (16%)	
1	0 (0%)	0 (0%)	1 (4%)	0 (0%)	0 (0%)	1 (2%)	
CSF ApoC-I (ng/ml)							0.731
Median (IQR)	106 (57)	96 (64)	153 (115)	169 (120)	200 (50)	158 (115)	
CSF ApoC-II (ng/ml)							0.650
Median (IQR)	15 (11)	25 (4)	21 (14)	19 (7)	15 (6)	20 (13)	
CSF ApoC-III (ng/ml)							0.804
Median (IQR)	109 (31)	81 (40)	69 (69)	76 (37)	67 (16)	79 (53)	
Plasma ApoC-I (μg/ml)							0.641
Mean (SD)	116 (12)	133 (15)	113 (22)	109 (20)	111 (21)	113 (20)	
Plasma ApoC-II (μg/ml)							0.320
Median (IQR)	6.73 (2.32)	10.37 (4.43)	7.02 (8.45)	7.71 (3.29)	12.40 (9.92)	7.36 (7.99)	
Plasma ApoC-III (μg/ml)							0.043^b^
Median (IQR)	198 (13)	161 (4)	107 (63)	136 (42)	136 (44)	130 (73)	

a no significant TukeyHSD pairwise comparisons

b significant TukeyHSD pairwise comparison for Ɛ3/Ɛ3- Ɛ2/Ɛ3 (*P* adj = 0.022)

### Apolipoproteins C-I and C-II truncations

**Fig. 1:**
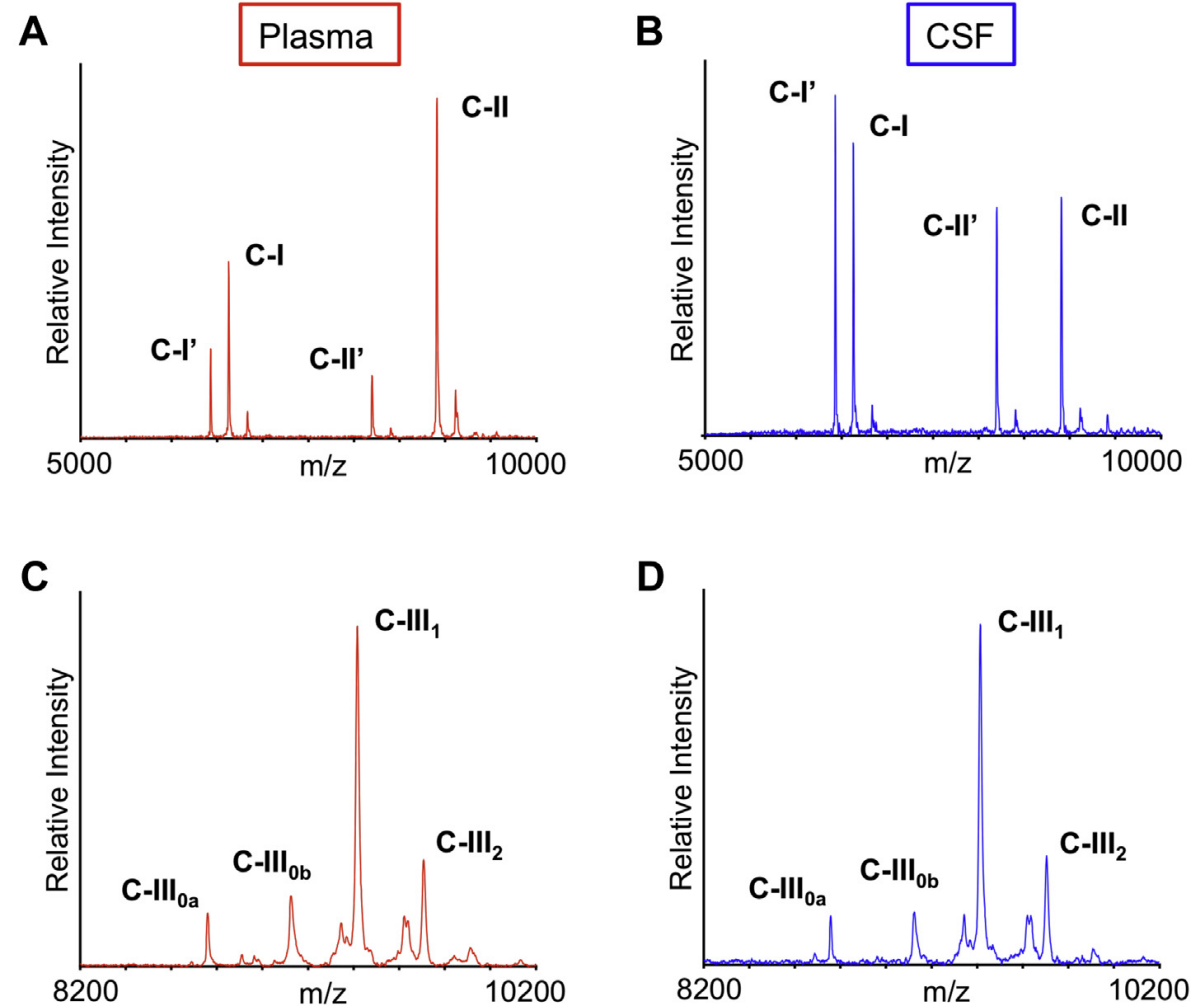
MALDI-TOF mass spectra for apoC-I and apoC-II resulting from the analysis of (A) plasma and (B) CSF paired samples obtained from a single individual. C-Iʹ, truncated apoC-I; C-IIʹ, truncated apoC-II. MALDI-TOF mass spectra for apoC-III resulting from the analysis of (C) plasma and (D) CSF paired samples obtained from the same individual. ApoC-III_0a_, no glycosylation; apoC-III_0b,_ GalNac-Gal; apoC-III_1,_ GalNac-Gal-Sia; apoC-III_2_, GalNac-Gal-Sia-Sia.

**Fig. 2:**
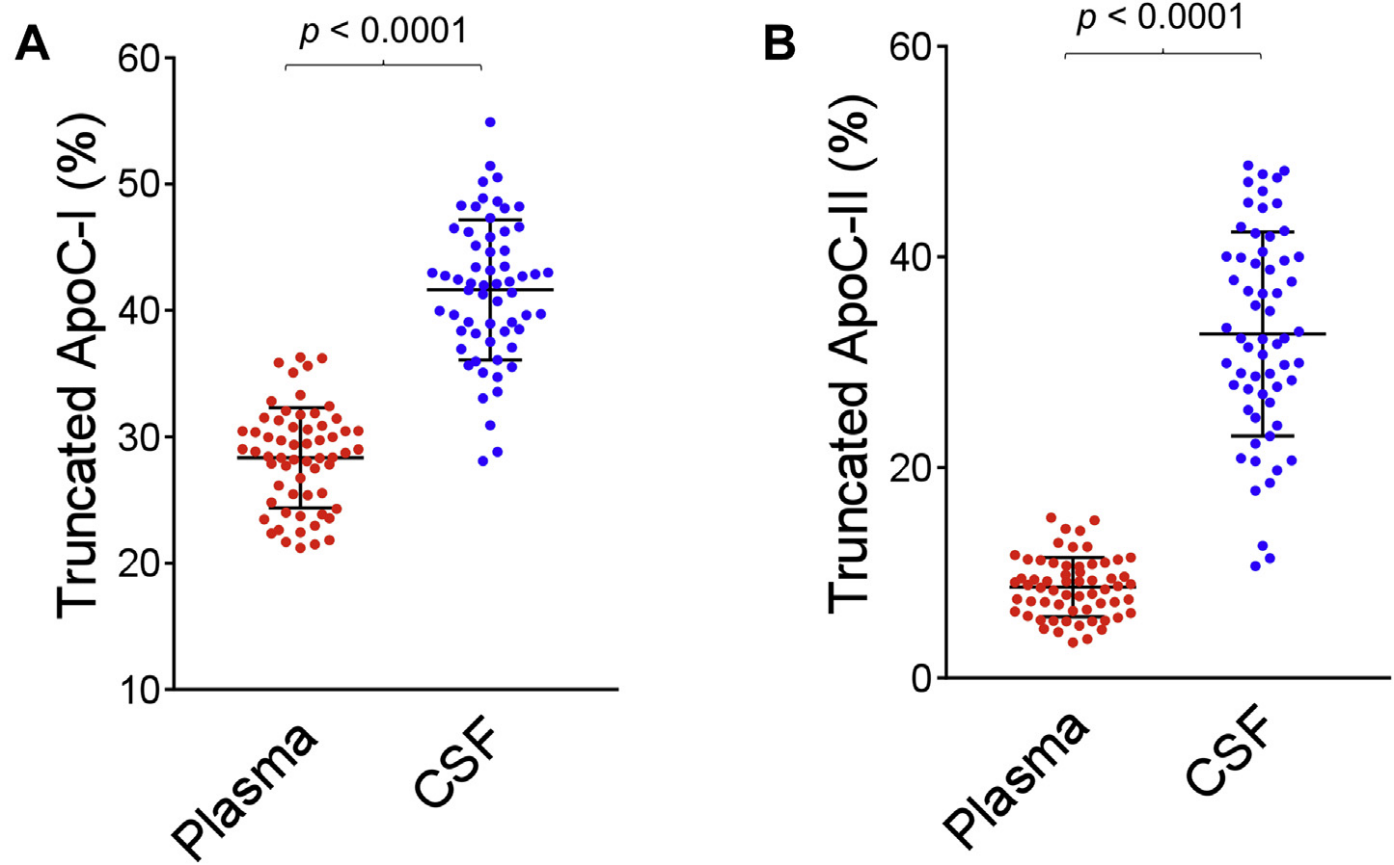
Percent abundance of (A) truncated apoC-I and (B) truncated apoC-II in plasma versus CSF. Percent truncation was computed by dividing the peak area of each truncated isoform with the summed peak areas of the truncated and full-length protein. Parametric paired *t*-tests were performed to identify differences between plasma and CSF samples between the normally distributed data sets.

**Fig. 3:**
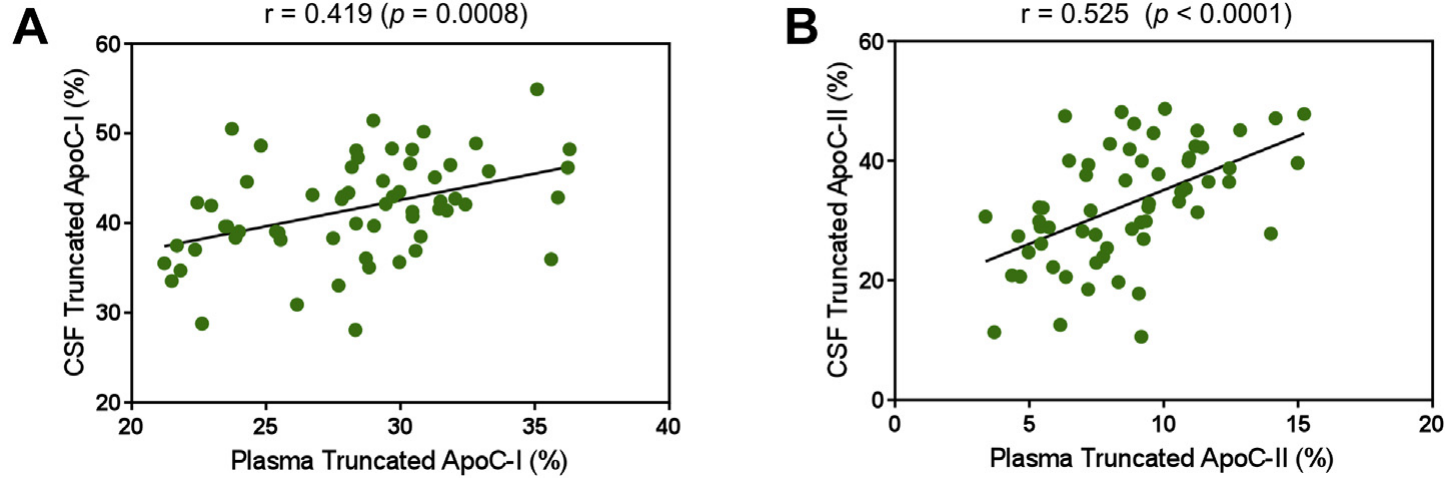
Correlation between (A) truncated apoC-I and (B) truncated apoC-II in the paired plasma and CSF samples. Shown are the parametric Pearson's correlation coefficients.

ApoC-I and apoC-II MALDI-TOF mass spectra resulting from the analysis of paired plasma and CSF samples from a single individual are shown in Fig. 1A, B. Strong signals for apoC-I (MW = 6630.58) and apoC-II (MW = 8914.92) are present in the mass spectra from both plasma and CSF, along with signals of their truncated isoforms (apoC-Iʹ MW = 6432.35 and apoC-IIʹ MW = 8204.17). Compared with the signals of the full-length proteins, the signals for the truncated isoforms are stronger in CSF than in plasma. The percent abundance for truncated apoC-I and apoC-II was obtained by dividing the peak area of the truncated isoform with the summed peak area of the truncated and full-length protein and is shown in Fig. 2 for all 61 plasma and CSF samples. Both proteins were truncated significantly more in CSF than in plasma. For truncated apoC-I, a mean of 41.6 ± 5.6% (SD) was observed in CSF compared with 28.3 ± 4.0% in plasma (*P* < 0.0001). For truncated apoC-II, a mean of 32.7 ± 9.7% was observed in CSF versus 8.62 ± 2.8% in plasma (*P* < 0.0001). The correlations of truncated apoC-I and apoC-II between plasma and CSF are shown in Fig. 3. A positive trend of increased truncations in CSF with increased truncations in plasma was observed, with Pearson correlation coefficients of r = 0.419 (*P* = 0.0008) for truncated apoC-I, and r = 0.525 (*P* < 0.0001) for truncated apoC-II. There was no significant correlation between the total concentrations of apoC-I and apoC-II and their respective truncated isoforms in both plasma and CSF, except for plasma apoC-II, with r = 0.352 (*P* = 0.013) (supplemental Fig. S1).

These results suggest increased proteolytic processing of apoC-I and C-II in CSF, creating more truncated isoforms. The enzyme responsible for removing the two N-terminal amino acids of apoC-I is dipeptidyl peptidase-IV (DPP-IV) (2). DPP-IV is expressed throughout the body, including the CNS (31, 32) where it participates in the regulation of biologically active peptides. ApoC-I is a suitable substrate peptide for DPP-IV (33), because it contains the preferred sequence NH_2_-Thr-Pro, and the N terminus is in a flexible conformation (34, 35). The higher abundance of truncated apoC-I in CSF may therefore indicate a higher level of DPP-IV expression/activity in the brain.

The enzyme responsible for the cleavage of apoC-II is unknown, but it has been speculated that it is the same endopeptidase that cleaves the six N-terminal amino acids of apoA-I (36), because the cleavage sites of the two proteins share sequence homology (37). Interestingly, while almost all of apoA-I in circulation is in the form of the truncated isoform (termed mature apoA-I), the truncated apoC-II constitutes only a minor fraction (< 10%) of total apoC-II in plasma (1, 3). The significantly increased percentage of truncated apoC-II in CSF may also indicate increased expression/activity of this enzyme, similar to DPP-IV.

The linear trend of increased truncations in CSF with increased truncations in plasma could also indicate a connection between the two pools of apoC-I and C-II in plasma and CSF. Expression of apoC-I in astrocytes and endothelial cells within the hippocampus and frontal cortex has been demonstrated (38, 39), but there is very little evidence for apoC-II expression in the brain. Thus, it is possible that some apoC-I and C-II may cross the BCB barrier and enter the CSF from plasma, most likely carried by the HDL, which crosses the barrier by transcytosis (40, 41). It is also possible that that transport of the truncated apoC-I and apoC-II isoforms across the BCB is increased compared with the full-length proteins.

The observed differences in apoC-I and apoC-II proteolytic cleavages between plasma and CSF may also be caused by the different types of lipoprotein particles in the periphery and CNS. In plasma, apoC-I and C-II are rapidly interchanged among HDL, CM, and VLDL and may be somewhat shielded from extensive proteolysis. In CNS, apoC-I and C-II are most likely only associated with HDL-like particles and may be more prone to proteolysis as they disassociate from the HDL. Therefore, domain interactions between phospholipids and bound apolipoproteins may protect them against proteolysis. It was, however, shown that lipid-free apoC-II's N and C terminals had a considerable percentage of shielding from hydrogen/deuterium (H/D) exchange and proteolysis (42), suggesting some structured order that helps protect against proteolysis. Increased levels of protease inhibitors in plasma should also be considered (43) and together may provide a more comprehensive explanation for the present trends. Further studies are warranted to explore these differences and delineate the functionality of the truncated isoforms of apoC-I and C-II.

### Apolipoprotein C-III isoforms

**Fig. 4:**
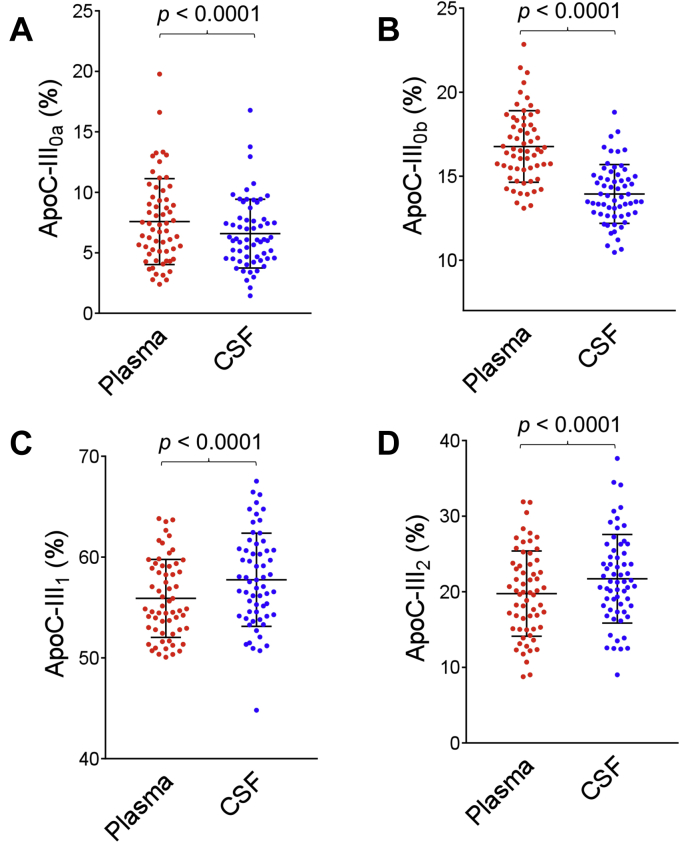
Percent abundance of: (A) apoC-III_0a_ (B) apoC-III_0b_ (C) apoC-III_1,_ and (D) apoC-III_2_ in plasma versus CSF. Percent abundance was computed by dividing the peak area of each isoform with the summed peak areas of all apoC-III isoforms. Parametric paired *t*-test was performed to identify differences between plasma and CSF samples for normally distributed data sets (apoC-III_0b_ and apoC-III_2_). Nonparametric Wilcoxon matched-pairs signed rank test was applied to data sets that were not normally distributed (apoC-III_0a_ and apoC-III_1_).

**Fig. 5:**
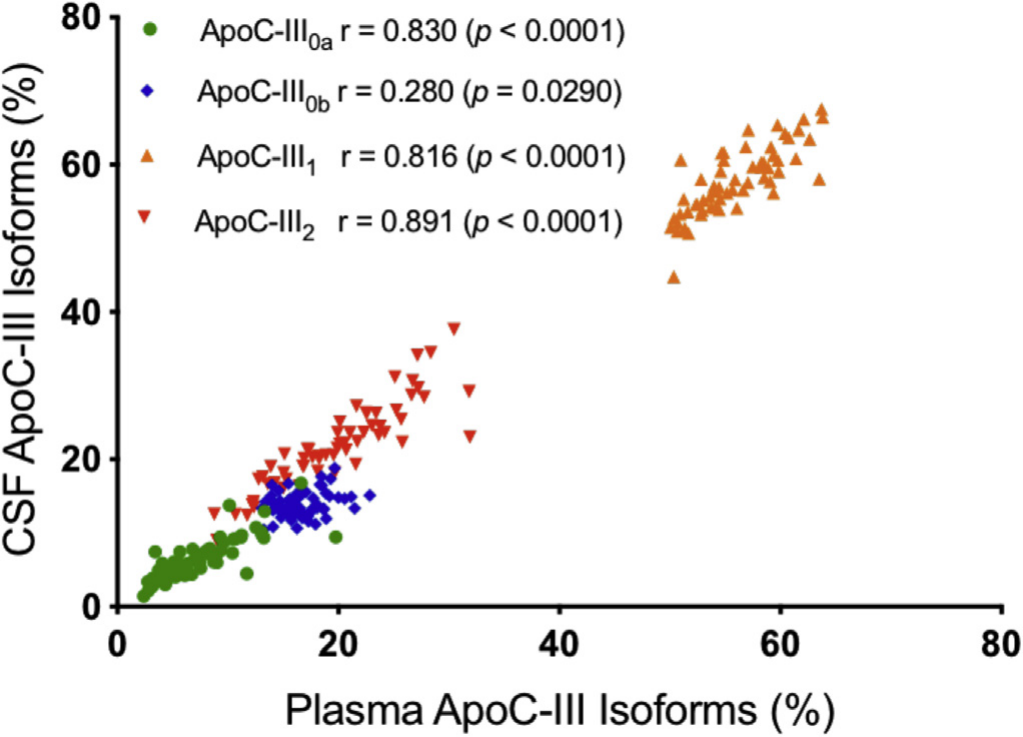
Correlation between apoC-III isoforms in the paired plasma and CSF samples. Shown are the parametric Pearson's correlation coefficients for apoC-III_0b_, apoC-III_2_, and the nonparametric Spearman correlation coefficients for apoC-III_0a_ and apoC-III_1_.

ApoC-III MALDI-TOF mass spectra resulting from the analysis of paired plasma and CSF samples from a single individual are shown in Fig. 1C, D. Signals were observed for all four major apoC-III isoforms in both plasma and CSF: apoC-III_0a_ (no glycosylation at Thr74, MW = 8764.7); apoC-III_0b_ (Thr74-GalNAc-Gal, MW = 9129.9), apoC-III_1_ (Thr74-GalNAc-Gal-Sia, MW = 9421.1), and apoC-III_2_ (Thr74-GalNAc-Gal-Sia-Sia, MW = 9712.4). The apoC-III_1_ signal was strongest in both plasma and CSF. The percent abundance of the individual apoC-III isoforms was calculated by dividing the peak area of each isoform with the summed peak areas of all the apoC-III isoforms and is shown in Fig. 4 for all 61 plasma and CSF samples. The percent abundance for apoC-III_0a_ and apoC-III_0b_ was decreased in CSF: a mean of 6.58 ± 2.8% in CSF versus 7.58 ± 3.5% in plasma was observed for apoC-III_0a_ (*P* < 0.0001), and a mean of 13.9 ± 1.7 in CSF versus 16.8 ± 2.1% in plasma was observed for apoC-III_0b_ (*P* < 0.0001). The opposite trend was detected for apoC-III_1_ and C-III_2_, with both isoforms showing increased percent abundance in CSF. A mean of 57.8 ± 4.6% in CSF versus 55.9 ± 3.9% in plasma was observed for apoC-III_1_ (*P* < 0.0001), and a mean of 21.7 ± 5.9% in CSF versus 19.7 ± 5.6% in plasma was observed for apoC-III_2_ (*P* < 0.0001). The relationships between the apoC-III isoforms in plasma and CSF are shown in Fig. 5, revealing positive linear relationships for apoC-III_0a_ (r = 0.830, *P* < 0.0001), apoC-III_0b_ (r = 0.280, *P* = 0.0290), apoC-III_1_ (r = 0.816, *P* < 0.0001), and C-III_2_ (r = 0.891, *P* < 0.0001). There was a correlation between the total plasma apoC-III concentrations and three of the apoC-III isoforms: apoC-III_0b_ and apoC-III_1_ showed strong positive correlations, whereas apoC-III_2_ exhibited significant negative association with total plasma apoC-III concentrations (supplemental Fig. S2). In CSF, the correlations were not statistically significant, although similar trends were noticeable (supplemental Fig. S3).

This is the first study of the apoC-III isoforms in CSF. Previously, it was shown that total apoC-III concentration in CSF is only a small fraction of that measured in plasma (11, 12). We confirmed these findings by determining in this study that the concentration of apoC-III in CSF was <0.1% of that in plasma (Table 1). There is currently no evidence that apoC-III is expressed in the CNS; thus, the apoC-III detected in CSF most likely originates from plasma. The excellent linear correlation observed between plasma and CSF apoC-III isoforms in this study suggests a connection between the two pools of apoC-III.

One mechanism for the transport of apoC-III from plasma to CSF could be on small HDL. The discoidal shape of apoA-I containing small HDL may promote its brain delivery, in contrast to the large spherical HDL particles that are lipid-rich and have lower BBB transport (44). ApoA-I protein is abundant in CSF, but there is no apoA-I mRNA expression in the brain (7, 10, 13, 45). ApoA-I is believed to enter the CSF via the choroid plexus (13). In contrast to individual apolipoproteins, small HDL particles carry a large number of apolipoproteins (46) that include apoCs and can confer pleiotropic effects, allowing brain access for these apolipoproteins. This is further supported by recent findings that plasma apoA-I-associated apoC-III correlated most strongly with CSF apoC-III (12), suggesting that plasma apoC-III crosses into CSF along with apoA-I on HDL.

The percent abundance of the two sialylated apoC-III isoforms (apoC-III_1_ and C-III_2_) was increased in CSF compared with plasma. In plasma, apoC-III is distributed among all classes of lipoproteins, while CSF apoC-III is most likely associated only with HDL-like particles. It is possible that apoC-III_1_ and apoC-III_2_ are preferentially bound to the HDL, which can shield them from degradation and thus increase their CSF abundance. Furthermore, HDL-bound apoC-III_1_ and apoC-III_2_ crossing over from plasma across the BCB barrier would result in their enrichment in CSF. Supporting this hypothesis are results from studies with apoE, which has the same O-linked glycan structure as apoC-III. It was shown that the removal of the apoE sialic acids decreased the binding of apoE to HDL in plasma, leading to impaired reverse cholesterol transport (47). But unlike apoC-III, apoE is also expressed in the brain, and its terminal sialic acids are critically involved in the formation of the CSF lipoprotein particles (48). It is possible that the apoC-III sialic acids play similarly important role in the CSF lipoproteins.

ApoC-III is known to inhibit the very low density lipoprotein receptor/low density lipoprotein receptor (VLDLR/LDLR), but was not shown to affect VLDLs' binding to LDL receptor related protein 1 (LRP1) (49, 50). The presence of one sialic acid on apoC-III enabled TRLs' clearance through LDLR and LRP1, while apoC-III_2_ was shown to be preferentially cleared by heparan sulfate proteoglycan (HSPG)-type receptors (26). These receptors are also present in the brain (8) and mediate pathways toward endocytosis and lipid catabolism. Since nonsialylated apoC-III functions to inhibit HSPG and LRP1, the presence of negatively charged mono/disialic acids may serve to nullify apoC-III's interaction with HSPG's or LDLR's/LRP's by orientating these lipoproteins in a way that promotes endocytosis. This could allow the lipoproteins with an affinity for these receptors, such as apoA-I and apoE, to readily bind to them (51, 52). Furthermore, an increased rate of HDL endocytosis by neuronal cells may optimize neuronal cell maintenance, given that lipid metabolism is virtually isolated in the CNS (53).

### Correlation of apoCs isoforms with *APOE* Ɛ4 allele and CSF Aβ42

The apoE phenotypes for the individuals that provided the 61 plasma and CSF samples were known from genotyping. Five allelic *APOE* combinations were present in the cohort: 5 Ɛ2/Ɛ3, 2 Ɛ2/Ɛ4, 27 Ɛ3/Ɛ3, 16 Ɛ3/Ɛ4, and 11 Ɛ4/Ɛ4. We investigated the correlations of the apoCs isoforms with the Ɛ4 allele, which is the strongest genetic risk factor for developing AD (54, 55, 56, 57). The samples were grouped into two groups: non-Ɛ4 (32 samples) and Ɛ4 (29 samples, heterozygous or homozygous for Ɛ4). ApoC-I and C-II truncationsand apoC-III isoforms percent abundance were compared between the two groups, in both plasma and CSF.

**Fig. 6:**
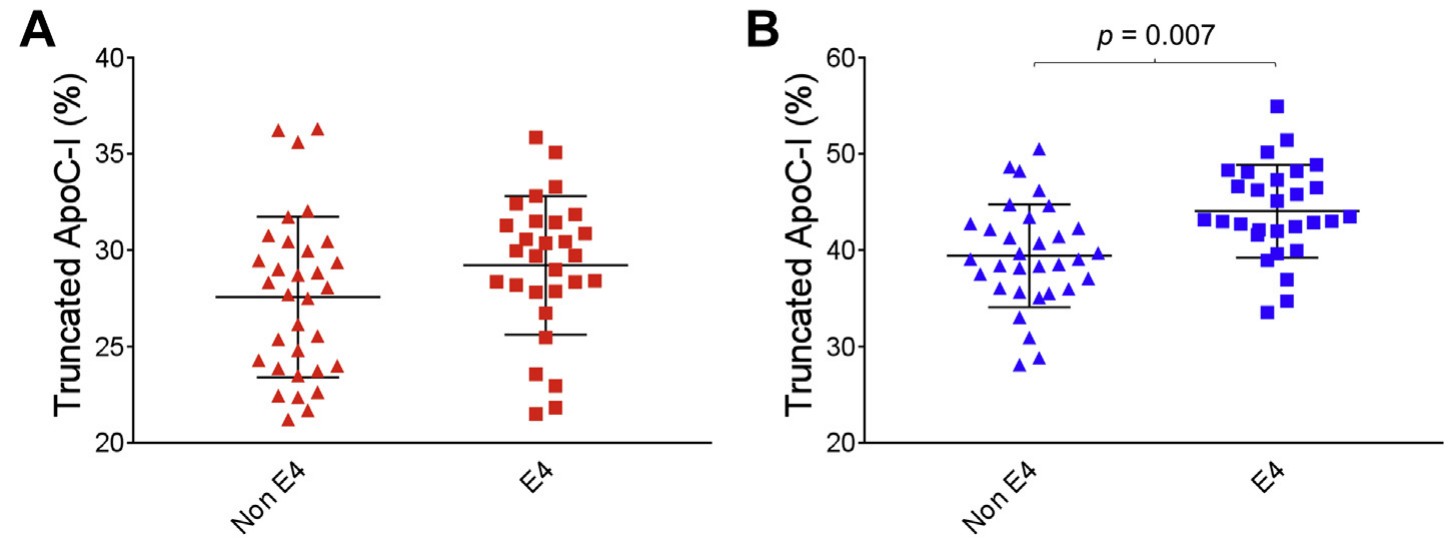
Percent abundance of truncated apoC-I in non-Ɛ4 versus Ɛ4 allele carriers, in (A) plasma and (B) CSF. Unpaired *t*-test with Welch's correction was utilized for the normally distributed data sets.

**Fig. 7:**
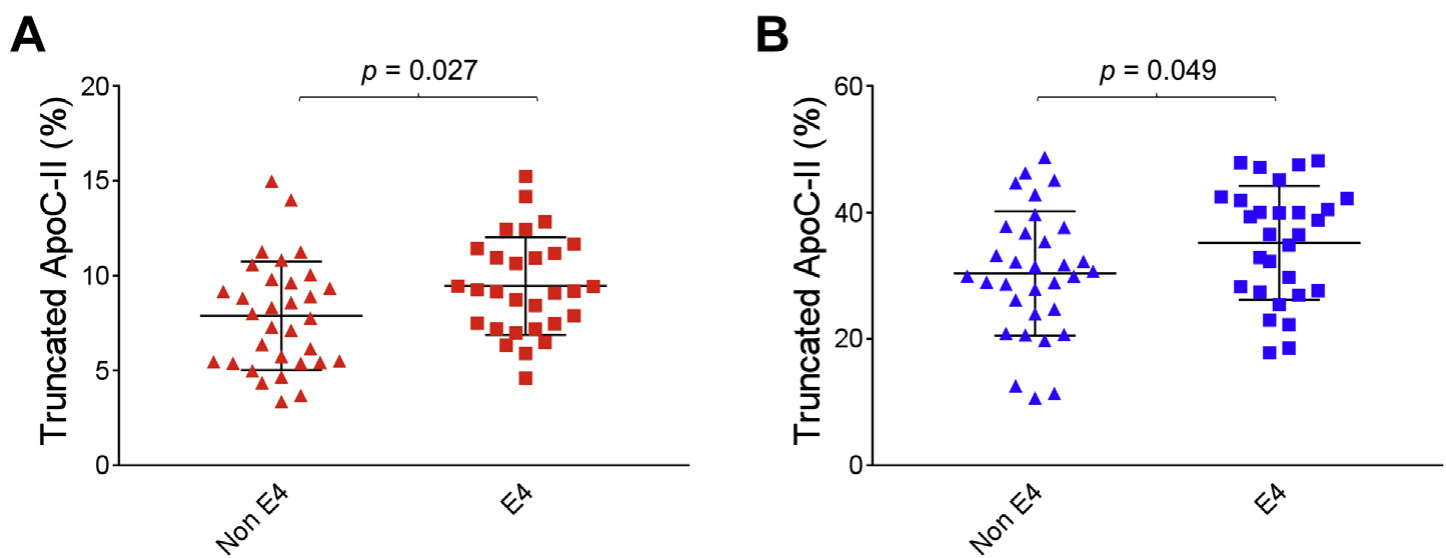
Percent abundance of truncated apoC-II in non-Ɛ4 versus Ɛ4 allele carriers, in (A) plasma and (B) CSF. Unpaired *t*-test with Welch's correction was utilized for the normally distributed data sets.

In plasma, the percent truncated apoC-I in the Ɛ4 group was 29.2 ± 3.6% versus 27.6 ± 4.2% in the non-Ɛ4 group (Fig. 6A). In CSF, the Ɛ4 group showed a significantly greater percentage of truncated apoC-I (44.1 ± 4.8%) when compared with the non-Ɛ4 group (39.4 ± 5.3%) (*P* = 0.007) (Fig. 6B). A statistically significant difference was also observed for the truncated apoC-II in both plasma and CSF, with the Ɛ4 group exhibiting higher percent truncation. In plasma, 9.45 ± 2.6% truncated apoC-II was observed in the Ɛ4 group versus 7.88 ± 2.9% in the non-Ɛ4 group (*P* = 0.027), while in CSF 35.2 ± 9.0% truncated apoC-II was observed in the Ɛ4 group versus 30.4 ± 9.9% the non-Ɛ4 group (*P* = 0.049) (Fig. 7). We also investigated the difference in apoC-I and apoC-II percent truncations between homozygous and heterozygous Ɛ4 carriers (supplemental Fig. S4). In plasma, these differences were not statistically significant, while in CSF only the truncated apoC-I exhibited similar differences between the homozygous Ɛ4 and the non-Ɛ4 group (*P* = 0.012) and the heterozygous Ɛ4 and the non-Ɛ4 group (*P* = 0.018). Total apoC-I and apoC-II concentrations were not significantly different among the various *APOE* genotypes, in both plasma and CSF (Table 1).

We also examined the association of the truncated apoC-I and C-II isoforms with CSF Aβ42, a surrogate of brain amyloid accumulation. There was a trend association between the % of truncated CSF apoC-I and CSF Aβ42 (r = −0.273, *P* = 0.055) and a significant association between truncated plasma apoC-II and CSF Aβ42 (r = −0.383, *P* = 0.006) (supplemental Fig. S5). This association persisted after adjusting for *APOE* genotype and did not differ between the two CDR groups (CDR = 0 and CDR > 0.5). Albeit modest, these associations may support a role of apoC truncations as biomarkers of brain amyloid plaques, reflecting a proteolytic milieu within the Aβ plaques that promotes greater enzymatic truncation of local (brain) or systemic apoC proteins. The nonsignificant associations of the other apoC-I and C-II truncations with CSF Aβ42 may be a function of the smaller sample size of this cohort.

**Fig. 8:**
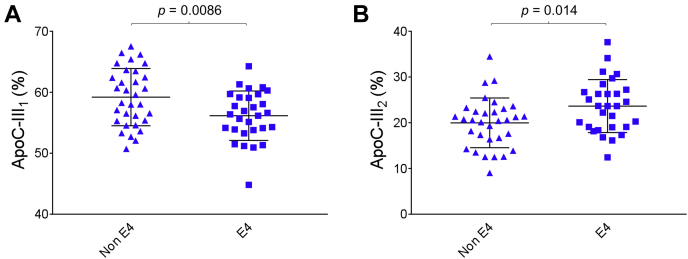
Percent abundance in CSF of (A) apoC-III_1_ and (B) apoC-III_2_ isoforms in non-Ɛ4 versus Ɛ4 allele carriers. Unpaired *t*-test with Welch's correction was utilized for the normally distributed data sets.

Of the four apoC-III isoforms, only apoC-III_1_ and C-III_2_ in CSF exhibited statistically significant differences between the Ɛ4 and non-Ɛ4 groups. The directions were opposite, with apoC-III_1_ decreased and apoC-III_2_ increased in the Ɛ4 group: 56.1 ± 4.1% apoC-III_1_ was observed in the Ɛ4 group versus 59.2 ± 4.7% in the non-Ɛ4 group (*P* = 0.0086); and 23.6 ± 5.8% apoC-III_2_ was observed in the Ɛ4 group versus 20.0 ± 5.4% in the non-Ɛ4 group (*P* = 0.014) (Fig. 8). The increased percent apoC-III_2_ suggests increased sialylation in the Ɛ4 group. ApoC-III_1_ and apoC-III_2_ did not show a statistically significant difference in plasma between the Ɛ4 and non-Ɛ4 groups, and neither did apoC-III_oa_ and C-III_ob_, in both plasma and CSF (supplemental Figs. S6 and S7). The difference in apoC-III isoforms between the homozygous and heterozygous Ɛ4 carriers in plasma was also not statistically significant (supplemental Fig. S8). In CSF, only the homozygous Ɛ4 carriers showed statistically significant difference from the non-Ɛ4 group for apoC-III_1_ (*P* = 0.033) and apoC-III_2_ (*P* = 0.020) (supplemental Fig. S9), suggesting that the observed differences between the Ɛ4 group and the non-Ɛ4 group shown in Fig. 8 were driven by the homozygous Ɛ4 carriers. The total apoC-III concentrations were not significantly different among the various *APOE* genotypes in both plasma and CSF (Table 1). Furthermore, there were no significant associations between the apoC-III isoforms and CSF Aβ42 (data not shown).

The interplay between apoE and apoCs is critical to the regulation of lipoprotein metabolism. The apoC-I and C-II genes are colocated with the apoE gene in a cluster on chromosome 19, and their expression is coregulated (5). In addition to the well-documented role of the *APOE* Ɛ4 allele as a risk factor for AD, apoC-I allelic variations were also suggested to be significant risk factor for AD (58, 59). ApoC-I was colocalized with amyloid-β (Aβ) in senile plaques from the brains of AD patients and shown to exacerbate soluble Aβ oligomer-induced neuronal death (39). Significantly reduced levels of ApoC-I mRNA were found in the frontal cortex of AD patients that carried the Ɛ4 allele (38), and apoC-I levels in CSF were found to vary with *APOE* genotype, with Ɛ4 carriers showing significantly less apoC-I (60). Increased truncation with the Ɛ4 allele was also observed with apoC-II, suggesting a common denominator to this phenomenon that could be modulated by the Ɛ4 allele and/or the expressed apoE4 isoform.

The truncations of apoC-I and apoC-II in the brain may reflect the plaque accumulation environment that is increased with APOE4. The emergence of protein deposits is linked to many pathologies like AD and CVD and therefore relevant to study. In general, the reactive oxygen species (ROS) (61, 62, 63, 64) produced from atherosclerotic and Aβ plaques may lead to a feedback loop that promotes apolipoprotein truncation (65). Indeed, apoE4 is more sensitive to proteolytic cleavage (66), and its truncated fragments have been found in the brains of AD patients (67). Specifically, apoE4[Δ(166–299)] was shown to promote the intracellular uptake of Aβ42, leading to an increased production of ROS (62). While it does play a critical role in AD pathogenesis, apoE4 was also shown to be associated with atherosclerosis (68). Therefore, there may be a correlation among some apolipoproteins and amyloid plaque formation. However, the biological route remains obscure, and whether increased proteolysis of related apolipoproteins is necessarily involved remains unclear. In a previous study, which demonstrated that some of the lipid-free apoC-II's amino acid sequences are protected from proteolytic cleavage, it was proposed that certain morphologies can promote amyloid formation: monomeric lipid-free apoC-II's regions that were less protected from H/D exchange and proteolysis corresponded to the core within its amyloid fibrils (42). Interestingly, apoC-II, apoA-I, and apoB aggregates were also found in atherosclerotic plaques (69), and apoA-I, apoA-II, apoC-I, apoB100, and apoE were found to colocalize with amyloid in vivo (39, 70, 71), although they were not necessarily truncated. The increased truncation of apoC-I and apoC-II in ε4 carriers and its relation to developing pathologies, such as AD, warrant further investigation.

While apoC-III's polymorphisms and relative abundance are affected by *APOE4* expression, the present ε4 trends in apoC-III sialylation are perhaps a reaction to ε4 pathology that affects lipid metabolism. The observed divergent tendencies of apoC-III_1_ and C-III_2_ with the Ɛ4 allele in CSF are intriguing and may be specific only to CSF as those differences were not statistically significant in plasma. It was observed previously that carriers of the Ɛ4 allele had increased apoC-III/apoE plasma ratios compared with the other two apoE alleles (72). Our previous plasma apoC-III isoforms studies revealed a negative relationship between apoC-III_2_ and TG, which was the opposite of the positive correlation observed for the other three isoforms (23, 24). We also showed that plasma apoC-III_2_ was preferentially cleared by HSPG type of receptors, whereas apoC-III_1_ was cleared more rapidly through LDLR and LRP1 (26). In another study, apoC-III_2_ demonstrated diminished ability to inhibit VLDL binding to the lipolysis-stimulated receptor in rat liver plasma membranes, unlike apoC-III_1_ which showed greater inhibitory effect (73), which might explain apoC-III_2_'s negative relationship with TG. In the current study, we demonstrate an increased percent abundance of apoC-III_2_ with the Ɛ4 allele in CSF. Based on the peripheral sink hypothesis, apoC-III on TG-rich lipoproteins can serve as a circulating Aβ binding protein that could facilitate the efflux of Aβ from the brain (74), and apoC-III sialylation may affect this process.

It has been shown that lipoprotein bound apoE4 does not preferentially bind to LRP1 like the other two apoE isoforms (E2 and E3), which was suggested to affect Aβ clearance (75, 76). Instead, Aβ and Aβ-apoE complexes are redirected to VLDLR in ε4 carriers and thus cleared at a much slower rate (77). This receptor also has a much slower endocytosis rate (78), which may affect clearance of HDL particles in the CNS. One possibility is that the increased presence of apoC-III_2_ within the CNS of Ɛ4 carriers facilitates endocytosis by offering an alternative route to HDL catabolism via HSPG type of receptors. Although apoE4 has been shown to bind with HSPG receptors with similar affinity as apoE3, it has also been demonstrated that apoE inhibits Aβ clearance by HSPG (79). Consequently, this would exacerbate Aβ accumulation as apoE and Aβ compete for HSPG type of receptors' binding. Therefore, the increased ratio of apoC-III/apoE in the HDL of ε4 carriers may serve to nullify this effect, allowing for increased Aβ clearance.

One of the limitations of our study is that the observed apoCs differences may result from a differential antibody-antigen capture from plasma and CSF. This is possible but less likely. The comparison within plasma and CSF by AD risk factors argues against this limitation. For example, the % apoCs truncation in APOE4 carriers was increased in both CSF and plasma (Figs. 6 and 7). Spike and recovery experiments were not feasible because apoCs standards commercially available do not contain the same isoforms (in the case of apoC-I and II) and are grossly oxidized (for all 3 apoCs), which creates additional signals in the mass spectra and makes the quantitative comparison using those standards inaccurate. For this assay, well-characterized plasma samples as QC points for all of our analyses, and not purified protein standards, were used.

A second limitation was that the study sample size was relatively small. This limited our ability to detect whether the % of apoC isoforms differed by early disease markers such as CDR 0 and CDR 0.5. Future studies will expand subgroup analysis to help understand the relationship of apoC processing with clinical disease onset.

## Conclusion

Distinct patterns of apoCs isoforms were detected in CSF in a set of paired plasma and CSF samples obtained from a cohort of healthy individuals. Truncated apoC-I and C-II isoforms were elevated in CSF, which could be the result of increased enzymatic activity. Sialylated isoforms of apoC-III were also elevated in CSF, possibly indicating preferred binding to HDL. Some of the apoCs isoforms' changes were accentuated for individuals that were carriers of the *APOE* Ɛ4 allele. ApoC-I and C-II truncations were greater in Ɛ4 carriers. The doubly sialylated apoC-III isoform was also elevated in Ɛ4 carriers, in agreement with previous observations in plasma about the distinctive feature of this isoform. Future studies should evaluate how the *APOE* Ɛ4 allele affects the apoCs metabolism and regulation, which may lead to implications on protein-related pathologies, such as AD and CVD.

### Data avilability

All data are contained within the manuscript.

## Conflict of interest

The authors declare that they have no conflicts of interest with the contents of this article.
