# Supplementary material for: Distinct patterns of apolipoprotein C-I, C-II, and C-III isoforms are associated with markers of Alzheimer’s disease
Source: J Lipid Res. 2020 Dec 18;62:100014. doi: 10.1194/jlr.RA120000919 (PMC7859854; doi:10.1194/jlr.RA120000919)
Supplement: Supplemental Figures S1 to S9 [file mmc1.pdf]

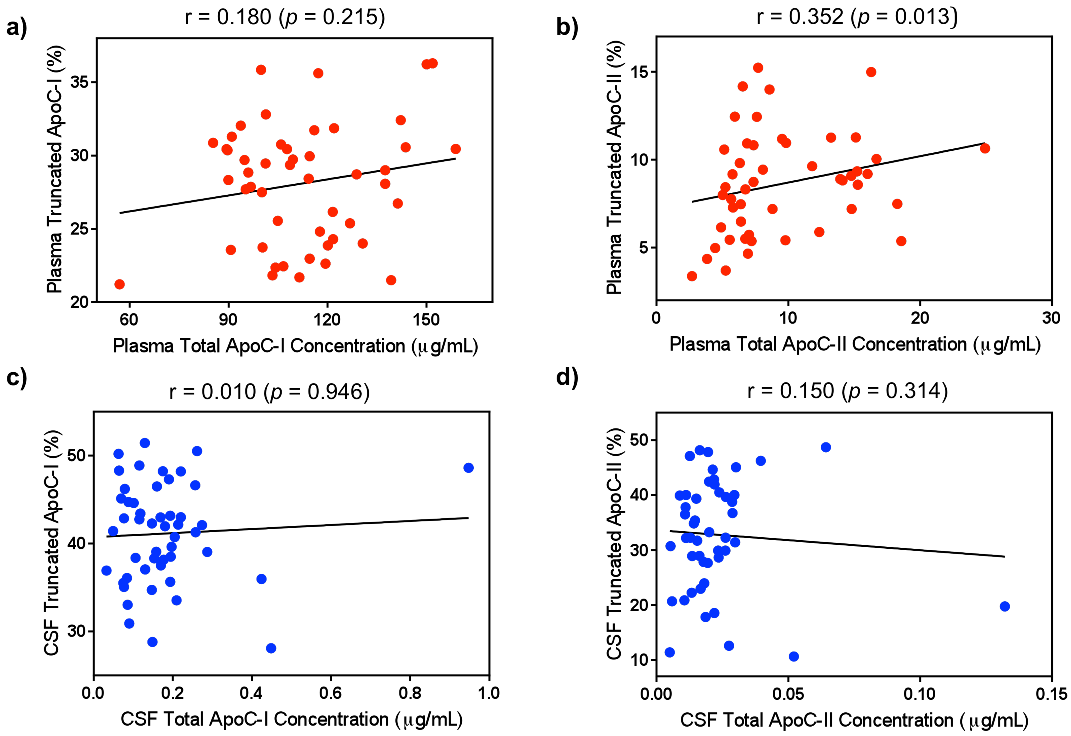

Supplemental Figure S1. Correlations between the total concentrations of apoC-I and apoC-II and their truncated isoforms. **a)** plasma apoC-I, **b)** plasma apoC-II, **c)** CSF apoC-I, and **d)** CSF apoC-II. Shown is the parametric Pearson's correlation coefficient for apoC-I in plasma, and the non-parametric Spearman's rank correlation coefficients for apoC-I in CSF, and apoC-II in plasma and CSF.

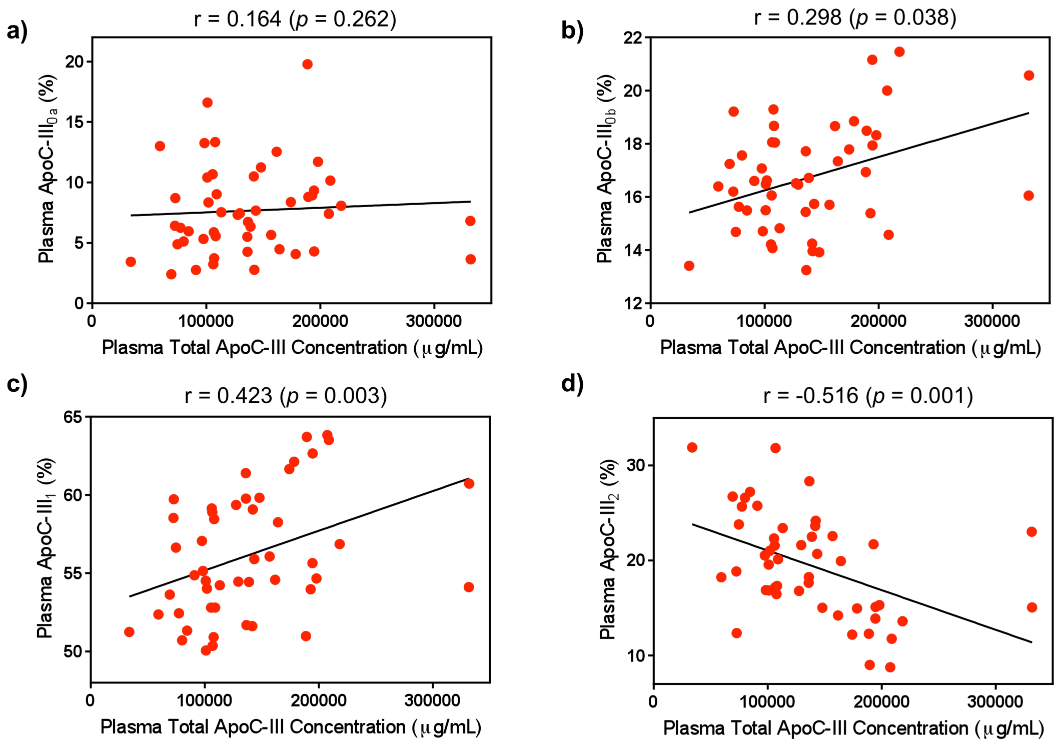

Supplemental Figure S2. Correlations between the total concentrations of apoC-III and its isoforms in plasma. **a)** apoC-III$_{0a}$, **b)** apoC-III$_{0b}$, **c)** apoC-III$_1$, and **d)** apoC-III$_2$. Shown are the non-parametric Spearman's rank correlation coefficients.

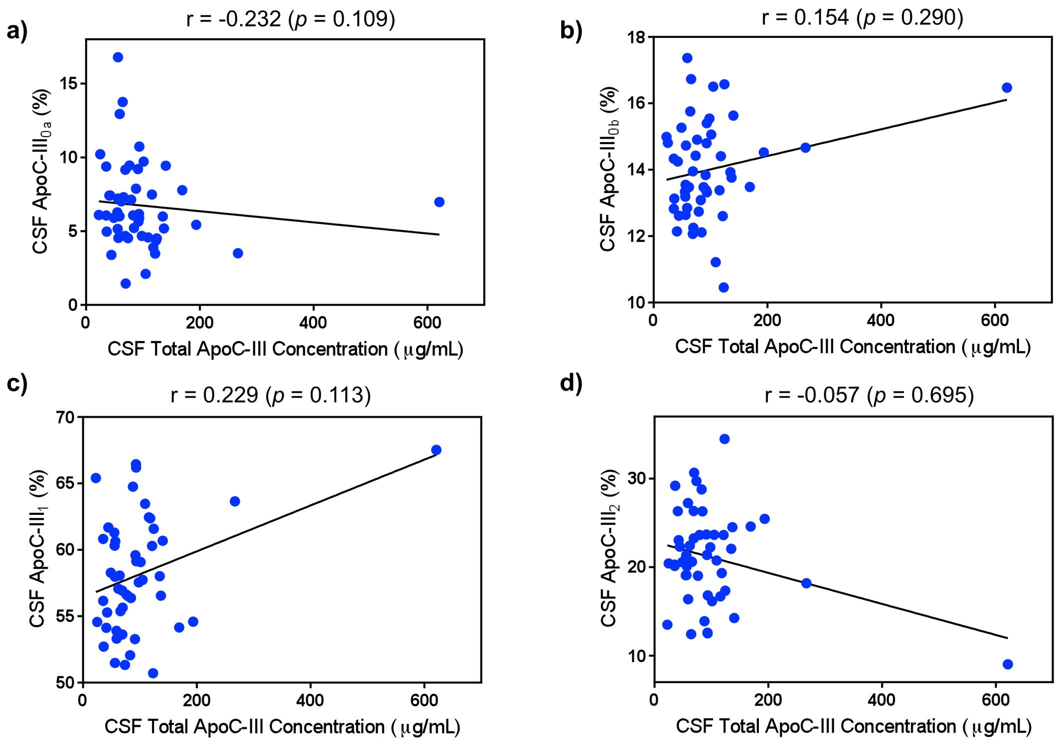

Supplemental Figure S3. Correlations between the total concentrations of apoC-III and its isoforms in in CSF. **a)** apoC-III$_{0a}$, **b)** apoC-III$_{0b}$, **c)** apoC-III$_1$, and **d)** apoC-III$_2$. Shown are the non-parametric Spearman's rank correlation coefficients. Correlations did not change significantly after removing the single outlier at > 600 μg/mL total apoC-III concentration.

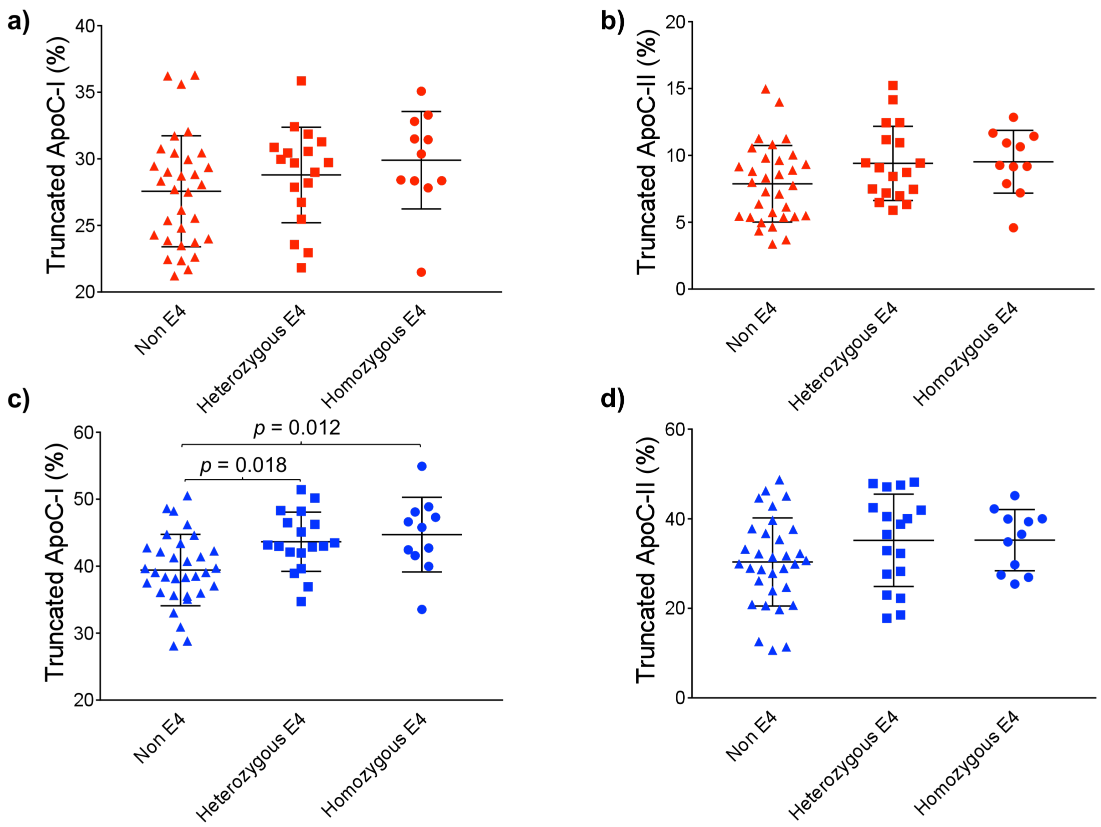

Supplemental Figure S4. Percent abundance of truncated apoC-I and apo-C-II isoforms among non-Ɛ4, homozygous Ɛ4, and heterozygous Ɛ4 allele carriers. **a)** Plasma apoC-I, **b)** Plasma apoC-II, **c)** CSF apoC-I, and **d)** CSF apoC-II. One-way ANOVA with *post-hoc* Tukey HSD test was performed at a 5% false discovery rate. Only statistically significant differences are shown ($p < 0.05$).

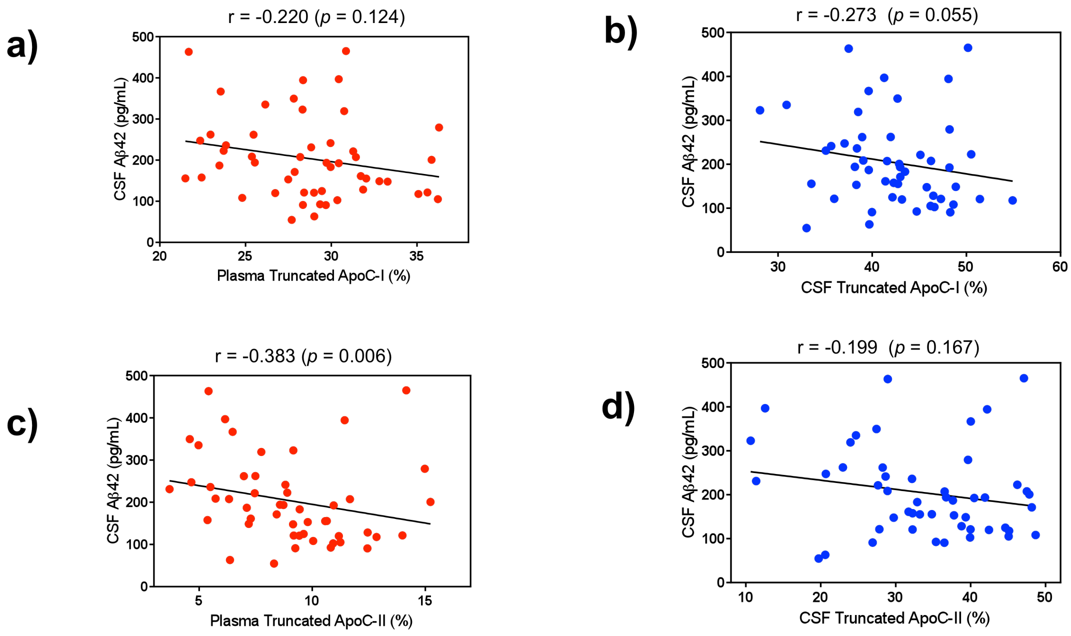

Supplemental Figure S5. Correlation between CSF Aβ42 and truncated apoC-I in **a)** plasma, and **b)** CSF samples; and between CSF Aβ42 and truncated apoC-II in **c)** plasma, and **d)** CSF samples. Shown are the non-parametric Spearman correlation coefficients.

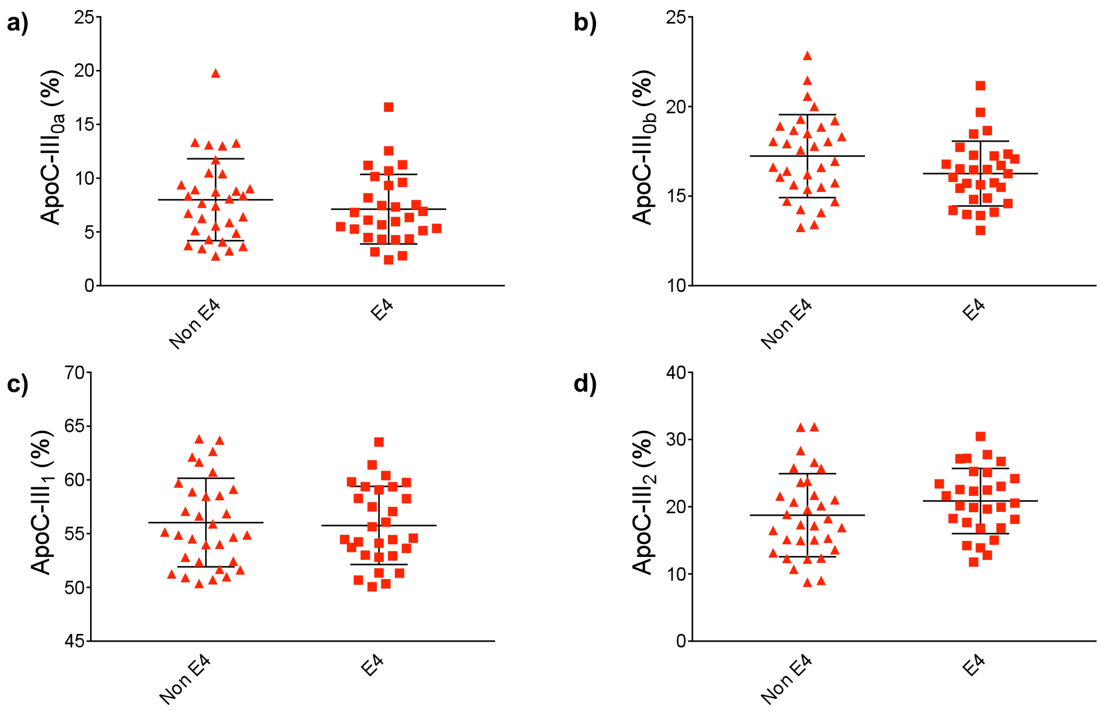

Supplemental Figure S6. Percent abundance of **a)** apoC-III$_{0a}$ **b)** apoC-III$_{ob}$ **c)** apoC-III$_1$ and **d)** apoC-III$_2$ isoforms in non-Ɛ4 vs. Ɛ4 allele carriers in plasma. Mann−Whitney test at 5% false discovery rate was applied to the non-normally distributed data apoC-III$_{0a}$ data set (p=0.39). Unpaired t-test with Welch's correction was utilized for the normally distributed data sets of apoC-III$_{ob}$ (p=0.071), apoC-III$_1$ (p=0.79), and apoC-III$_2$ (p=0.14).

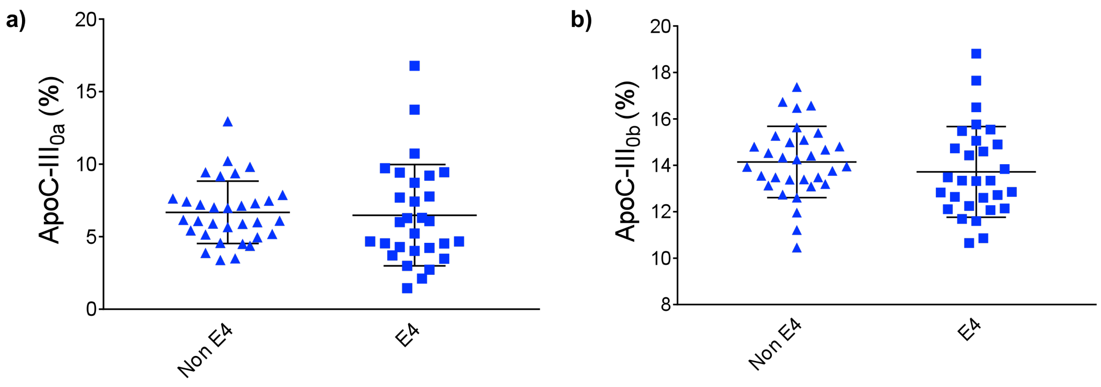

Supplemental Figure S7. Percent abundance of **a)** apoC-III_{0a} and **b)** apoC-III_{0b} isoforms in non-Ɛ4 vs. Ɛ4 allele carriers in CSF. Mann−Whitney test at 5% false discovery rate was applied to the non-normally distributed data apoC-III_{0a} data set (p=0.45). Unpaired t-test with Welch's correction was utilized for the normally distributed data set of apoC-III_{ob} (p=0.35).

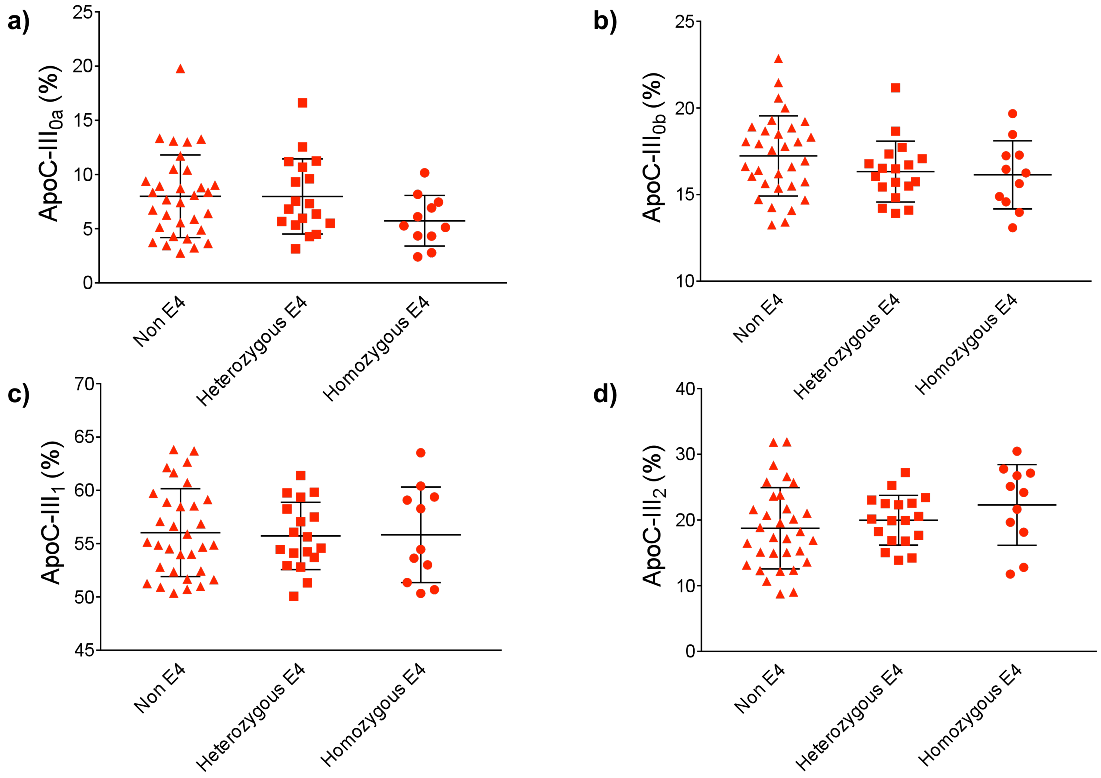

Supplemental Figure S8. Percent abundance of truncated apoC-III isoforms in plasma among non-Ɛ4, homozygous Ɛ4, and heterozygous Ɛ4 allele carriers. **a)** apoC-III$_{0a}$, **b)** apoC-III$_{0b}$, **c)** apoC-III$_{1}$, and **d)** apoC-III$_{2}$. Kruskal-Wallis test with Dunn's multiple comparisons test was applied to the non-normally distributed data apoC-III$_{0a}$ data set. One-way ANOVA with *post-hoc* Tukey HSD test was perfomed at a 5% false discovery rate for the normally distributed data sets of apoC-III$_{ob}$, apoC-III$_{1}$, and apoC-III$_{2}$. None of the differences were statistically significant ($p > 0.05$).

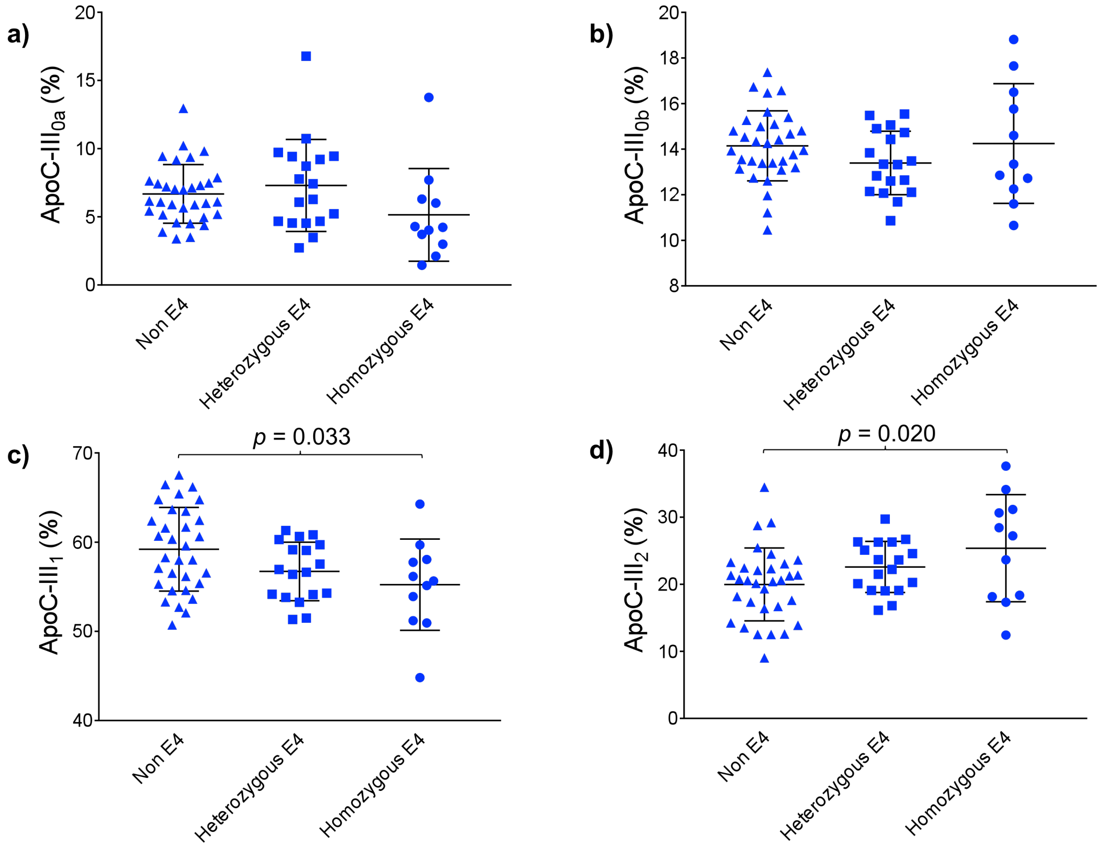

Supplemental Figure S9. Percent abundance of truncated apoC-III isoforms in CSF among non-Ɛ4, homozygous Ɛ4, and heterozygous Ɛ4 allele carriers. **a)** apoC-III_{0a}, **b)** apoC-III_{0b}, **c)** apoC-III_1, and **d)** apoC-III_2. Kruskal-Wallis test with Dunn's multiple comparisons test was applied to the non-normally distributed data apoC-III_{0a} data set. One-way ANOVA with *post-hoc* Tukey HSD test was perfomed at a 5% false discovery rate for the normally distributed data sets of apoC-III_{0b}, apoC-III_1, and apoC-III_2. Only statistically significant differences are shown ($p < 0.05$).
